# Potential of Biocellulose Carrier Impregnated with Essential Oils to Fight Against Biofilms Formed on Hydroxyapatite

**DOI:** 10.1038/s41598-018-37628-x

**Published:** 2019-02-04

**Authors:** Adam Junka, Anna Żywicka, Grzegorz Chodaczek, Mariusz Dziadas, Joanna Czajkowska, Anna Duda-Madej, Marzenna Bartoszewicz, Katarzyna Mikołajewicz, Grzegorz Krasowski, Patrycja Szymczyk, Karol Fijałkowski

**Affiliations:** 1 Department of Pharmaceutical Microbiology and Parasitology, Wrocław Medical University, Borowska 211A, 50-556 Wrocław, Poland; 2 Department of Immunology, Microbiology and Physiological Chemistry, Faculty of Biotechnology and Animal Husbandry, West Pomeranian University of Technology, Szczecin, Piastów 45, 70-311 Szczecin, Poland; 3 Laboratory of Confocal Microscopy, Polish Center for Technology Development PORT, Stablowicka 147, 54-066 Wrocław, Poland; 4 Faculty of Chemistry, University of Wroclaw, Joliot-Curie 14, 50-353 Wrocław, Poland; 5 Laboratory of Microbiology, Polish Center for Technology Development PORT, Stabłowicka 147, 54-066 Wrocław, Poland; 6 Department of Medical Microbiology, Wroclaw Medical University, Chałubińskiego 4, 50-534 Wrocław, Poland; 7 Nutrikon, KCZ Surgical Ward, Krakowska 32A, 46-020 Opole, Poland; 8 Centre for Advanced Manufacturing Technologies (CAMT/FPC), Faculty of Mechanical Engineering, Wrocław University of Science and Technology, Łukasiewicza 5, 50-371 Wrocław, Poland

## Abstract

In this research, bacterial cellulose (BC), one of the most promising biopolymers of the recent years, was saturated with thyme, eucalyptus and clove essential oils (EOs) and applied against staphylococcal and pseudomonal biofilms formed on hydroxyapatite (HA). BC dressings were thoroughly analyzed with regard to their physical properties. Moreover, the exact composition and ability of particular EO molecules to adhere to HA was assessed. Additionally, cytotoxicity of oil-containing, cellulose-based dressings towards osteoblasts and fibroblasts as well as their impact on reactive oxygen species (ROS) production by macrophages was assessed. The results revealed the high ability of BC dressings to absorb and subsequently release EOs from within their microstructure; the highest number of compounds able to adhere to HA was found in the thyme EO. The eucalyptus EO displayed low, while thyme and clove EOs displayed high cytotoxicity towards fibroblast and osteoblast cell lines. The clove EO displayed the highest eradication ability toward staphylococcal, while the thyme EO against pseudomonal biofilm. Taken together, the results obtained indicate the suitability of EO-saturated BC dressings to eradicate pseudomonal and staphylococcal biofilm on HA surface and moreover, to not trigger reactive oxygen species production by immune system effector cells. However, due to cytotoxic effects of thyme and clove EOs towards cell lines *in vitro*, the eucalyptus EO-saturated BC dressing is of highest potential to be further applied.

## Introduction

A biofilm is a community of microbial cells embedded within an extracellular matrix of various chemical and structural composition. From the clinical point of view, the most important biofilm features include its high resistance to antimicrobials and immune system together with high ability to colonize patients’ tissues and virtually all medical devices and equipment that stays in contact with water or water-based liquids. The recognition of biofilm’s ubiquity in nosocomial settings resulted in re-shaping the dogmas of clinical microbiology and approaches in the context of infection prophylaxis and treatment^1^.

Microbial biofilm tolerance to antimicrobials depends on the type of the extracellular matrix and on biofilm-forming species themselves (including intrinsic and/or acquired resistance mechanisms) and, last but not least, on the biofilm location within the patient’s body. Our recent findings and the results of teams dealing with the diagenesis phenomenon indicate that biofilms are able to re-model bone structures and penetrate within this tissue, thus significantly hampering antibiotic penetrability within the infection site^2,3^. One effective strategy to fight bone biofilm is to provide high local concentration of antimicrobial to the direct proximity of the bone using such antimicrobial carriers as chitosan, hyaluronic acid or collagen^4^. Among the flaws of a majority of such natural carriers are: low stability, insufficient mechanical strength and hard-to-standardize pore size/space^5,6^. These disadvantages may cause disruption as well as uncontrolled content release from a carrier. Therefore none of the aforementioned carriers meets all the requirements for a so-called “ideal carrier” which constitutes a powerful mixture of such features as low cytotoxicity and allergenicity, high release potential and appropriate mechanical strength^7^.

The bacterial cellulose (BC) polymer produced by such non-pathogenic bacterial species as *Komagataeibacter xylinus*^8^ is presently considered an ideal wound dressing, meeting all the requirements of a modern wound dressing material. It has been used increasingly frequently for chronic traumas regardless of their etiology^9–11^. Therefore, we assumed that such BC’s properties as non-toxicity, non-allergenicity, high tensile strength, flexibility and great compatibility with living tissues but also high water holding capacity and a pronounced permeability would be of great utility^9,12^ especially considering the reports on cost-effective ways of producing this polymer^12,13^.

However, one should notice that BC, which basically is a biofilm matrix to *K*. *xylinus* microbes, obviously does not possess antimicrobial properties. Therefore, to equip a BC carrier with antimicrobial properties, the polymer should be impregnated with antimicrobial agents^13,14^. It has already been confirmed that BC can be effectively impregnated with various forms of active silver; antiseptics (including benzalkonium chloride, octenidine or polyhexanidine) and antibiotics (e.g. erythromycin)^15–17^.

It should be mentioned that the application of antiseptics directly onto the bone is forbidden due to high cytotoxicity of these agents towards bone-forming cells^18^ and possible systemic adverse reactions. Therefore, local application of high concentrations of antibiotics is commonly used in the clinical setting, however some promising trials on systemic use of quinolone hybrids were performed already on animal model^19^.

The above-mentioned issues together with increasing microbial resistance to antibiotics used in bone treatment (gentamycin, ciprofloxacin, rifampicin) prompted researchers to explore alternative new key molecules against bacterial species forming bone biofilm, with special stress placed on their anti-staphylococcal and anti-pseudomonal activity^20^.

Many of such molecules may be potentially found in essential oils (EOs). These aromatic oily liquids are derived from such materials of natural origin as flowers, buds, seeds, leaves, twigs, bark, herbs, wood, fruits and roots. There is plenty of evidence of EOs antimicrobial activity already^21–25^. Thanks to their hydrophobic composition, EOs actively bind to bacterial cell and mitochondrial membranes, disturbing the cell structures, increasing its permeability, which somewhat resembles the mechanism of action of many antiseptics^15,26^.

Although the use of BC as a drug delivery system and EOs as natural antimicrobial agents have recently attracted much interest there is a lack of reports describing the possibility of combining the favorable features of both of them to fight bone biofilm. Therefore, the aim of the current study was to combine BC and EOs (obtained from cloves, eucalyptus, thyme) into prototypical dressing designed to eradicate biofilm from hydroxyapatite (main inorganic bone component) *in vitro*.

## Results and Discussion

### Characterization of cellulose membranes

The ubiquitous prevalence of biofilms in nosocomial settings together with an alarming increase in microbial resistance to antibiotics have accelerated the search for new types of drugs and their suitable carriers. Herein we present data concerning the applicability of BC as a carrier for EOs – antimicrobials with potential to fight against biofilm-based bone infections.

**Figure 1:**
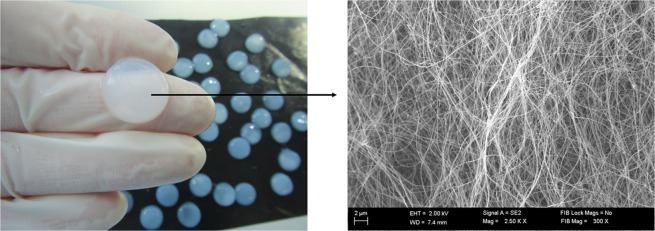
Microfibrilar structure of BC after biosynthesis. Mag.300x. SEM Zeiss Auriga 60.

Bacterial cellulose demonstrates unique properties, including high mechanical strength, high water holding capacity and good biocompatibility^14,27^. For this reason, BC biopolymer is considered presently a game-changer in the context of local treatment of biofilm-based infections^28^. In terms of structure, BC is composed of nanofibrils which subsequently crystallize into microfibrils. The microfibers of BC are ribbon-like structures of around 100 nm in diameter and around 100 µm long. These ribbons are made up of bundles of cellulose nanofibres of 2–4 nm in diameter^29^. The BC microfibrils are extruded by the bacterial cell and then they self-assemble to form a three-dimensional network structure resembling a sponge^10,30^. SEM analysis of a BC sample derived from *K*. *xylinus* strain used in this work confirmed a coherent 3-D network formed by the cellulose fibers (Fig. 1).

The porosity of the biomaterial is one of its key features with regard to tissue engineering, including bone implant application^10,31^. The analysis of the BC membrane used for impregnation with EOs showed that it displays an average surface area of 9.56 ± 0.83 m^2^/g, average pore volume of 3.42 ± 0.04 cm^3^/g and average pore diameter of 0.24 ± 0.01 nm. Several other research teams confirmed that the highly porous BC structure is one of the greatest advantages of this biomaterial when used for such medical applications as impregnation with active substances^20,27,32^.

The BC pellicles used in this study for impregnation displayed a swelling ratio of 353 ± 12.32% and a water holding capacity of 68 ± 4.83%. The ability to keep moisture within the proximity of bone surfaces provides an appropriate environment for proper osteoblast growth and multiplication, thus enabling effective repair and regeneration processes. According to the literature, the swelling capacity of unmodified BC is in the range of 200–500%, whereas the water holding capacity is between 50–100%^29,33,34^. Therefore, the water-related parameters of BC used in this study fit within the above-given range. Having the suitability of BC as a carrier proven we have subsequently analyzed the properties of EOs in the context of their suitability for bone biofilm eradication.

### Essential oils

All antimicrobials and antiseptics tested as active substances of BC dressings display some disadvantages which limit their usability in the prevention and treatment of biofilm-based bone infections. Therefore, in the present study we tested the activity of substances presently beyond strict clinical use with regard to bone treatment, namely clove (**C**), eucalyptus (**E**) and thyme (**T**) EOs as active substances of BC dressings. The rationale behind it was to provide an alternative for the already existing antimicrobials used to limit or eradicate bone infections, as their application correlates with the increasing resistance of microbes and subsequent therapeutic failures. In the first stage of our line of investigation, we determined the composition of the above EOs. The major substances detected are presented in Table 1. Additionally we evaluated which active substances from individual EOs adhere to hydroxyapatite and therefore may be used as future drugs for the treatment of bone infections.

**Table 1:** List of compounds found in clove, eucalyptus and thyme EOs including compounds adhered to hydroxyapatite surface.

Clove oil	Eucalyptus oil	Thyme oil
δ-cadinene	α-pinene	β-pinene
caryophyllene oxide	1,3-cyclohexadiene, 1-methyl-4-(1-methylethyl)-	3-carene
eugenol acetate*	trans-β-ocimene	ɣ-terpinene
**isocaryophyllene**	ɣ-terpinene	cyclohexene, 1-methyl-4-(1-methylethyl)-
eugenol*	β-ocimene	antar
caryophyllene*	cyclohexene, 3-methyl-6-(1-methylethylidene)-	cyclohexene, 1-methyl-4-(1-methylethylidene)-
	eucalyptol*	camphene
	β-pinene*	**1**,**3**,**8–menthatriene**
	***o*** -cymene*	δ-limonene*
	δ-limonene*	isoborneol*
		eucalyptol*
		**caryophyllene oxide**
		**linalol**
		**cyclohexanol**, **1-methyl-4-(1-methylethenyl)-**
		**bicyclo[7**.**2**.**0]undec-4-ene**, **4**,**11**,**11-trimethyl-8-methylene-**,[1r-(1r*,**4z**,9 s*)]-
		caryophyllene*
		isoborneol*
		terpineol*
		**π-menth-8-en-1-ol**, **stereoisomer**
		thymol*
		**3-cyclohexen-1-ol**, **1-methyl-4-(1-methylethyl)-**
		**camphor**
		**linalyl acetate**
		**terpinen-4-ol**

Compounds adhered to HA discs with preformed biofilm on them are distinguished with bold font. Compounds adhered to HA discs and of known antimicrobial activity are distinguished with asterisks.

EOs listed in Table 1 are known for their antimicrobial potential; however, they are composed of a plethora of substances which may or may not be active against microorganisms. Therefore, there is a threat that the interactions between some of them diminish their antimicrobial activity due to antagonistic interaction or some of ingredients may even stimulate microbial growth. Therefore, we have tested which molecules of EOs adhere to hydroxyapatite, because only such molecules may be applied specifically as drugs to combat biofilms adhered to the bones. It occurred that only some of them are able to do so. The majority of compounds found to be bound to HA, e.g. eugenol, eucalyptol, thymol, isoborneol, caryophyllen, cymene, pinene or limonene are also compounds of proven antimicrobial activity^21–25^. Moreover, an interesting trend was observed – the higher the number of specific types of antimicrobial molecules found adhered to the HA surface (Table 1, molecules distinguished with an asterisk), the higher the biofilm eradication ratio was observed (please refer to *Anti-biofilm activity analysis of BC dressings against biofilmic forms of pathogens in an environment simulating bone conditions* section). This trend was particularly well-visible in the case of staphylococcal biofilm but not so distinct for pseudomonal biofilm, probably due to generally weaker EO activity against Gram-negative than against Gram-positive pathogens proven by Nazzaro *et al*.^35^. The majority of the above-mentioned antimicrobials present low solubility in water^36–38^. Therefore, it can be assumed that only their combined activity could lead to the observed antibiofilm effect and the more types of molecules were involved, the stronger it was.

### Analyses related to impregnated membranes

**Figure 2:**
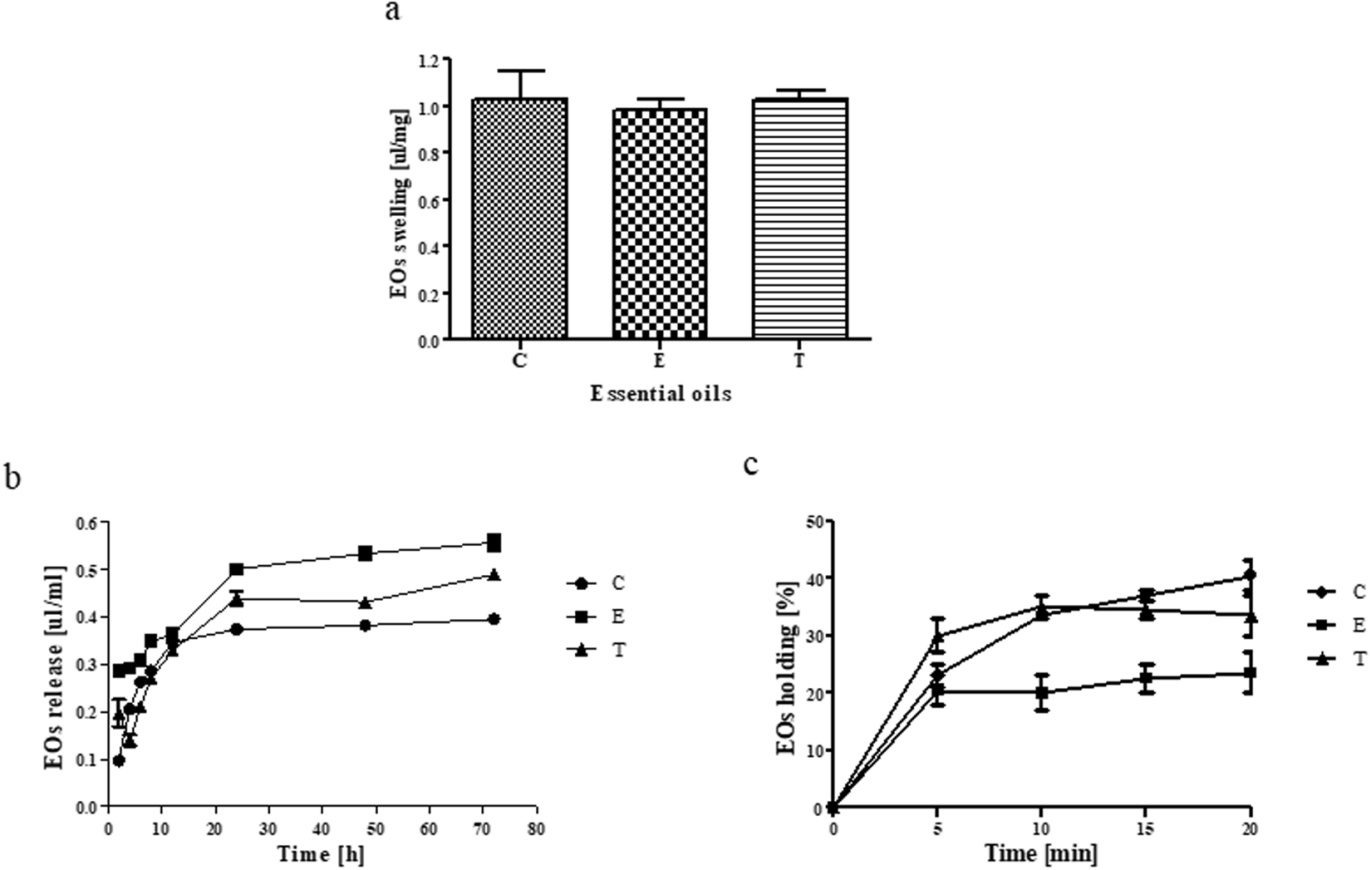
The effectiveness of impregnation process: (**a**) BC swelling capacity of Eos. (**b**) EOs release ratio from BC. (**c**) EOs holding capacity (EOHC). The data are presented as a mean ± standard error of the mean. Results of swelling capacity represent accumulated release. C - clove oil, E - eucalyptus oil, T - thyme oil. No significant differences between particular EOs were found (K-W test, p < 0.05).

The nanoporous structure of BC and its high swelling ability allows not only to saturate and release drugs, but also serves as an efficient physical barrier sequestrating the microbes within. It was shown that dry BC was able to absorb 0.98–1.03 µL of oils/mg of dry BC (Fig. 2a). No significant differences between individual EOs absorbed volume were observed (K-W test, p < 0.05).

In the case of prevention against orthopedic infections, not only the working concentration of the antimicrobial released from a carrier, but also its release time is of key significance^34^. A lasting antimicrobial effect requires a continued release of the antimicrobial agent from the biomaterial in effectively working concentrations^9^. The slow release of the antibacterial substance from BC was observed by Morritz *et al*.^16^ as well as by Wei *et al*.^27^ who tested octenidine dihydrochloride and benzalkonium chloride, respectively. The above-mentioned researchers attributed it to the unique fibrillar nanostructure and three-dimensional network of this material. Therefore, we analyzed the release behavior of EOs molecules from the BC pellicle as well as EOs holding capacity. The results of our study revealed that EOs molecules were released gradually from the BC pellicle (Fig. 2b,c). After 72 h of incubation on average 0.39–0.55 µL/ml of EOs was released from BC, which constituted about 47% of the initial amount of adsorbed oil. There were no significant differences between the release ratios of individual EOs absorbed (K-W test, p < 0.05). It should be emphasized that the high porosity of BC does not cause water or drug leakage from an impregnated membrane. Also, the holding capacity of the EO-impregnated BC was high. The main limitation of this experimental section was the fact that the analysis was performed in DMSO-containing environment because of slight water solubility of EOs analyzed. However, a comparative analysis of data from Fig. 2b and Table 1 indicates that the antimicrobials from EOs were able to get released from the BC disc and able to reach the HA structure. An extrapolation of data obtained from Fig. 2 and Table 1 into clinical conditions may allow to assume that the unique three-dimensional porous BC nanostructure ensured an effective level of EO absorption and their controlled long-term release^9,30,32^.

### Antibacterial activity of impregnated BC

**Figure 3:**
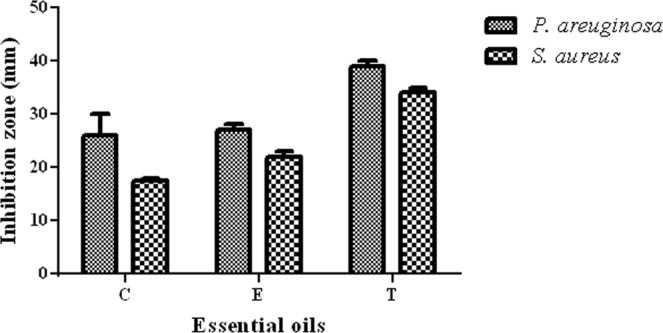
Means of inhibition growth diameter obtained by disc diffusion method (mm). Data are expressed as inhibition zone (mm) and presented as a mean ± standard error of the mean. C - clove oil, E - eucalyptus oil, T - thyme oil. No significant differences between inhibition zone for clove oil, eucalyptus oil and thyme oil were found (K-W test, p < 0.05).

The results of antimicrobial activity of EO-impregnated BC evaluated by the disc diffusion method are shown in Fig. 3. It was found that all impregnated BC pellicles exhibited antagonistic activities against *S*. *aureus* and *P*. *aeruginosa*. The size of *S*. *aureus* inhibition zones caused by the EOs released from BC discs was higher than for *P*. *aeruginosa* inhibition zones. The application of BC as an EO carrier corresponded to inhibition zones of a size comparable to the zones obtained by well diffusion method (no carrier) and higher than when a sterile filter paper disc was used as a carrier **(**Table 2 and Fig. S1**)**.

**Table 2:** Means of inhibition growth diameters obtained by diffusion methods (mm).

EOs Carrier	*P*. *aeruginosa*			*S*. *aureus*		
	BC	No carrier	Paper discs	BC	No carrier	Paper discs
C	27.0 ± 0.5	27.5 ± 1.5	24.0 ± 0.0	35.5 ± 1.5	37.0 ± 0.0	25.0 ± 0.0
E	25.5 ± 1.5	29.5 ± 0.5	22.5 ± 0.5	36.5 ± 0.5	37.5 ± 0.5	24.5 ± 0.5
T	35.0 ± 0.5	38.0 ± 0.0	32.5 ± 1.5	38.0 ± 0.0	40.0 ± 0.0	33.0 ± 0.0

BC - BC membrane impregnated with EOs; C - clove oil, E - eucalyptus oil, T - thyme oil. Data are expressed as inhibition zone (mm) and presented as a mean ± standard error of the mean. The volume of the EOs used was the same for all three methods and it was estimated on the basis of results obtained in Fig. 2a for a cellulose membrane.

Subsequently, we have performed a more elaborate analysis (A.D.A.M. test) aimed to show EOs penetrability through physical barriers and also, we performed an analysis of EOs antimicrobial activity in conditions simulating bone conditions.

**Figure 4:**
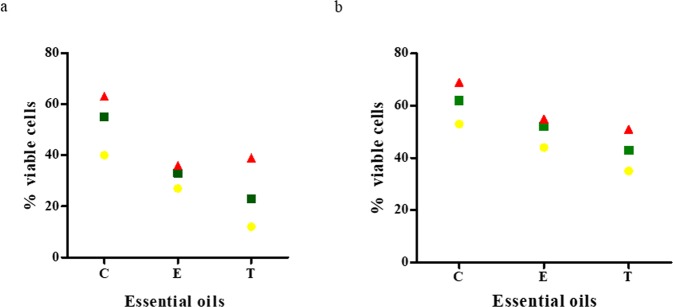
Reduction of *S*. *aureus* (**a**) and *P*. *aeruginosa* (**b**) biofilmic cells number as a result of EO-saturated BC activity. Red triangles - contact discs; green squares - media discs; yellow circles - bottom discs. The reduction ratio is normalized with regard to biofilm growth control samples which were estimated as a 100% of possible absorbance. C - clove, oil, E - eucalyptus oil, T - thyme oil.

The performance of the A.D.A.M. test allowed to estimate both the efficacy and the penetrability of the EOs released from the dressings (Fig. 4a,b and Table 3). Varying efficacy of the antimicrobials released from the dressings was observed depending on all analyzed variables, i.e. disc type (C, M, B), species of biofilm-forming pathogens, type of oil applied. Clove oil proved to be the least efficient against *S*. *aureus* and *P*. *aeruginosa* biofilm, while E and T EOs displayed the higher and comparable activity against these pathogens.

**Table 3:** Penetrability Index [PI] values and penetrability of BC impregnated with EOs.

Essential oil	Agar disc	*S*. *aureus*			*P*. *aeruginosa*		
		% Erad.	PI value	P	% Erad.	PI value	P
C	C	40	0.525	S	42	0.507	S
	M	27			40		
	B	12			38		
E	C	55	0.099	S	33	0.099	S
	M	33			32		
	B	23			27		
T	C	63	0.537	S	25	0.346	S
	M	36			20		
	B	39			14		

P - penetrability, H, S, M - high, strong, moderate respectively. C - clove oil, E - eucalyptus oil, T - thyme oil. % Erad. - percentage biofilm eradication. C - contact disc, M - middle disc, B - bottom disc.

Interestingly, according to the formula provided by A.D.A.M. test, namely the penetrability index [PI], all EOs analyzed displayed high penetrability within the subsequent biofilm matrices [PI < 1] with eucalyptus oil demonstrating the highest penetrability among other EOs applied.

### Anti-biofilm activity analysis of BC dressings against biofilmic forms of pathogens in an environment simulating bone conditions

**Figure 5:**
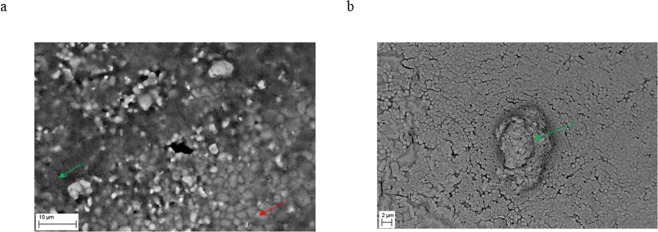
Confirmation of the ability of the tested staphylococcal strain (**a**) and tested pseudomonal strain (**b**) to form biofilm on HA structure. Native structure of hydroxyapatite (red arrow) and multilayer structure of biofilm (green arrows) are marked. Magn. 947x and 5000x, respectively. ZEISS EVO MA SEM.

To additionally test the ability of EOs released from BC to reach the biofilm formed on hydroxyapatite, we placed HA discs with pre-formed biofilm on it at the bottom of a 24-well plate. To simulate bone conditions, we incubated biofilm at 37 °C/5% CO_2_ in a medium for osteoblast culturing. To confirm biofilm presence on the HA discs we have performed SEM analysis. Both tested strains formed strong, multi-layer biofilm structure on the HA surface (Fig. 5).

**Figure 6:**
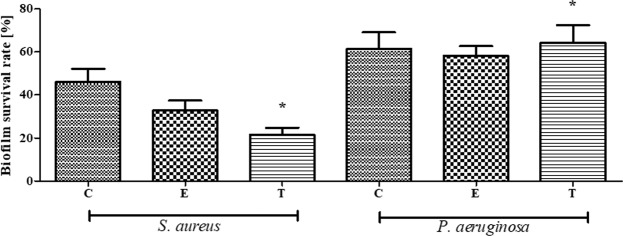
Biofilm Survival Rate [%] of *S*. *aureus* and *P*. *aeruginosa* biofilm on HA discs incubated in the presence of BC dressings impregnated with C - clove oil, E - eucalyptus oil, T - thyme oil. The results are normalized with regard to CP sample which consists of cellulose and PEG but does not possess active substances of EOs * - statistically significant differences between individual EOs and various bacteria (K-W test, p < 0.05).

Interestingly, contrary to our assumptions, biofilm eradication rate was for this experimental setting higher than in the case of results obtained in A.D.A.M. test (Fig. 6). It suggests that physical obstacles used in A.D.A.M. test were of more impeding nature for EOs than water-soluble components used in the latter analysis. Moreover, the biofilm grown in bone-simulating conditions was formed by a lower number of CFUs (data not shown) than in the A.D.A.M. test which could have an impact on the final outcome of the analysis. Regardless of above, the trend for biofilm eradication was similar in the context of EOs types applied – the clove oil displayed the lowest, while thyme oil the highest ability to eradicate the *S*. *aureus* biofilm. As regards biofilm formed by *P*. *aeruginosa*, all tested oils displayed very low efficacy in this setting, reaching ca. 30% of biofilm-forming cell reduction in comparison to a positive control setting. Additionally, it was found that the difference between the ability of thyme oil to reduce staphylococcal vs. pseuodomonal biofilm was statistically significant (K-W test, p < 0.05).

**Figure 7:**
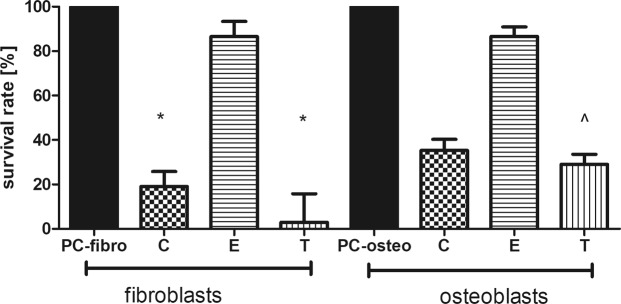
Survival Rate [%] of fibroblasts and osteoblasts incubated in presence of extracts from BC dressings impregnated with C - clove oil, E - eucalyptus oil, T - thyme oil. Results are normalized with regard to CP sample which consists of cellulose and PEG but does not possess active substances of EOs *statistically significant fibroblast cell number reduction between settings where EO-BC was applied vs. CP (K-W test, p < 0.05); ^statistically significant osteoblast cell number reduction between settings where EO-BC was applied vs. CP (K-W test, p < 0.05).

**Figure 8:**
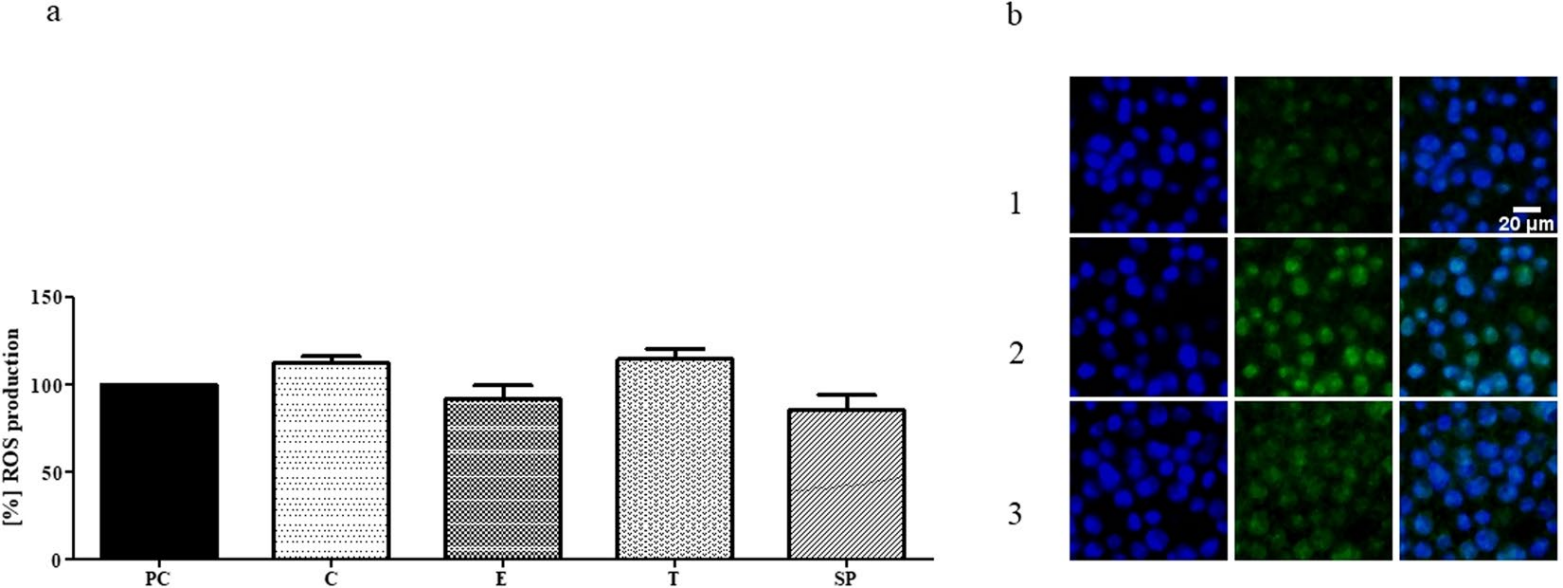
(**a**) Macrophage ROS production in the presence of extracts from BC dressings impregnated with PEG - PC; clove oil - C, eucalyptus oil - E, thyme oil - T. SP - spontaneous ROS production by non-treated macrophages. (**b**) Visualization of macrophage ROS: 1. spontaneous (non-treated) production; 2. LPS-induced ROS production; 3. ROS production induced by extract from BC dressing impregnated with eucalyptus essential oil. Green color - ROS production; blue color - macrophages’ nuclei. Please note that intensity of fluorescence of EO-treated macrophages (at green channel, especially) is similar to this of control sample and lower than of LPS-induced production. Control set: Results are normalized with regard to CP sample which consists of cellulose and oil PEG but does not possess active substances of EOs (values of ROS production for CP was considered 100%).

Cytotoxicity of EO-soaked BC tested against osteoblast and fibroblast cell cultures is presented in Fig. 7 and Table S1. It was found that the lowest cytotoxicity (the highest survival rate) was displayed by eucalyptus oil, while high cytotoxicity toward osteoblasts and fibroblasts was displayed by clove and thyme oils. EDX analysis showed that the observed cytotoxic effect is non-related to the presence of bacterial cell wall residues within the BC membrane (Fig. S2). Moreover, no cytotoxic effect was observed when the extract from bacterial cellulose supplemented with glycol (positive control) was applied. It means that clove and thyme EOs but not the BC carrier were responsible for the cytotoxic effects presented in Fig. 7 and Table S1. The presence of numerous components in the analyzed EOs (please refer to Table 1) that simultaneously interfere with the cell multiple signaling pathway^39^, might be the key reason to understand the observed differences in the survival rate of osteo- and fibroblast cell lines subjected to thyme, clove and eucalyptus essential oil activity (Fig. 7). As eucalyptus oil displayed high anti-biofilm activity as shown in Figs 3, 4 and 6, this type of EO should be considered the most suitable for use as bone biofilm eradicator. Moreover, also the measurement of reactive oxygen species [ROS] produced by macrophages in the presence of EOs-impregnated BC dressings revealed that eucalyptus oil displayed the lowest potential related with ROS production by macrophages among other EOs tested. However, this trend was not statistically significant (K-W test, p < 0.05) (Fig. 8).

It should be noted that eucalyptus oil does not possess strong antioxidative properties itself (please refer to Table 4), so the results presented in Fig. 8 showing a weak macrophage response to this particular oil are not biased by the masking effect of radical scavenging displayed by other oils analyzed.

**Table 4:** Antioxidant effectiveness of extracts obtained from BC impregnated with essential oils.

	BC-C	BC-T	BC-E	Medium
RS%	61.64 ± 2.72	46.10 ± 1.32	1.54 ± 0.03	0.80
Amount of oil	0.22 ± 0.01	0.27 ± 0.00	0.70 ± 0.01	—
RSC%	53.37 ± 3.24	31.65 ± 1.87	1.05 ± 0.07	0.33
Amount of oil	0.45 ± 0.01	0.48 ± 0.05	0.89 ± 0.02	—

RS - Radical scavenging, RSC - Radical scavenging capacity, BC-C - extract from BC impregnated with clove oil, BC-T - extract from BC impregnated with thyme oil, BC-E - extract from BC impregnated with eucalyptus oil. The amount of EOs in the extract based on the standard curve was calculated and is presented as mg of EOs/ml of extract.

These results suggest that the use of eucalyptus EO in BC carrier may be useful in the case of biofilm-based bone infection, where a massive release of ROS by macrophages (in response to the presence of a pathogenic biofilm) is attributed to the chronicity of biofilm-based infections.

A further optimization of antimicrobial-effective, but not cytotoxic concentrations of eucalyptus EO as an active ingredient of BC dressing may lead to its use as an efficient counter-measure against bone biofilm infections. Bone infection is a rare but extremely serious condition. Independently of the route of infection – microbial spread through the blood system, fracture or surgery – it unconditionally requires the application of antimicrobial countermeasures, including a surgical bone cleansing from necrotic and biofilm structures. In this article we provide *in vitro* data on the applicability of EO-containing BC dressings to fight against biofilm formed on hydroxyapatite. The next line of this investigation undoubtedly requires genotoxic tests and animal studies, but in the era of increasing antibiotic resistance, if further developed, such approach as herein presented may provide – an efficient clinical alternative for the presently used treatments, and therefore it is worth of continuation.

## Materials and Methods

### Biosynthesis of cellulose membranes

The reference strain of *Komagataeibacter xylinus* (Deutsche Sammlung von Mikroorganismen und Zellkulturen - DSM 46602) was used for the production of BC. To obtain BC pellicles of a regular shape and of equal diameter, *K*. *xylinus* was cultivated in stationary conditions in a 24-well culture plate (Becton Dickinson and Company, USA) for 7 days at 28 °C. The culture was conducted in the Herstin-Schramm (H-S) medium. The obtained BC pellicles were purified by treatment with 0.1 M NaOH at 80 °C for 90 min to remove the bacterial cells and medium components. The purified BC samples were washed until neutral pH value of washing water was reached, dried at 60 °C overnight and sterilized in an autoclave. The average diameter of BC pellicles used for impregnation was 1.5 cm. The average weight of a BC pellicle was approximately 0.90 g and 0.008 g for wet and dry BC, respectively.

### Characterization of the cellulose membranes

The obtained cellulose membranes were further evaluated with a set of analytical methods. The analyses included: microstructure evaluation using Scanning Electron Microscopy (SEM), porosity, pore size and surface area assessment with the use of N_2_, adsorption/desorption measurements and water related properties assessment. A detailed description of the aforementioned methods can be found in our earlier study^31^. The main aim of the physicochemical analysis of carrier properties was to control the parameters relevant to the application of BC as a dressing. The element composition of BC membranes was analyzed by means of Electron Dispersive Spectroscopy (EDX) to search for nitrogen and phosphorus remains. The performance of the EDX method is discussed in detail in our other study^40^.

### Essential oils

For impregnation of BC membrane, three EOs of confirmed or alleged antimicrobial activity obtained from clove, eucalyptus, thyme (PharmaTech, Poland) were used. Undiluted EO volumes were used for BC impregnation purposes. The composition of the EOs used in this study was analyzed using GC/MS coupled with mass spectrometer single quadrupole (Shimadzu, Japan) with split/splitless injector at 280 °C, transfer line 300 °C, ion source 240 °C, capillary polar column ZB-WAX 30 m × 0.25 mm × 0.25 I.D.; Phenomenex, USA). The analyses were performed after previous dilution in dichloromethane (1:50). Injection 1 µL was made in split mode (1:50). The oven program was: 40 °C – 1 min, 10 °C/min to 180 °C, 25 °C/min to 300 °C hold 5 min. The spectrometer was set to scan mode 10 000 scan/sec in the range 33–488 amu. The mobile phase was helium 99.999% (LindeGas, Poland) with linear velocity of 30 cm/sec in constant flow mode. The spectra were analyzed using GCMS Solution ver. 4.1 software (Shimadzu, Japan) with library Nist ver. 14 (NIST, USA).

### Impregnation process

For impregnation, BC pellicles were immersed in 5 mL of EOs for 24 h at room temperature in tightly closed container to prevent evaporation. Next, the BC pellicles were removed from the EOs and wiped with filter paper to remove non-absorbed substance. As a negative control, pure cellulose without immobilized oil was used. The BC negative control sample was wiped with filter paper in the same way as the sample investigated, i.e. the one used for impregnation.

### Analyses relating to the impregnated membranes

#### EO swelling capacity

Following impregnation, the BC samples were weighed using an analytical balance. Taking into account the weight of dry and impregnated BC (swollen BC), the amount of adsorbed EOs based on the standard curve was calculated. The results were presented as: µL of EOs/mg of dry BC. The experiment was conducted in technical triplicates and repeated three times.

#### EOs release rate

To assess the release of the tested antimicrobials from the BC discs, the spectrometric method was applied. Pellicles of impregnated BC were incubated for 72 h at 37 °C in sealed beakers containing 5 mL of dimethyl sulfoxide (DMSO, Sigma-Aldrich, Germany) EOs solvent. Each day, 200 μL of the obtained solution was transferred into wells on UV transparent 96-well microtitre plate (Becton Dickinson and Company, USA). During the first 12 h, measurements were performed every 2 h, and after this time, every 12 h. The absorbance was measured spectrometrically at 300 nm using microplate reader (Infinite 200 PRO NanoQuant, Tecan, Switzerland). Each time after absorbance reading, the sample taken for the measurements was re-set into the beaker. The experiment was conducted in technical triplicates and repeated three times.

The absorbance values obtained were used to calculate the concentration of EO released (µl/ml). The calculation was performed based on the standard curve with known concentrations of EOs (absorbance vs. EO concentration).

#### EO holding capacity

In the oil holding study, after the impregnation process, the BC pellicles were wiped carefully with filter paper, placed on an analytical balance (accurate to 0.0001 g), weighed and then centrifuged 2,000 RPM for 5 min x 5. Every 5 min, BC sample was weighed. EO holding capacity values were calculated using the formula (1):

1 $${\rm{EOs}}\,{\rm{holding}}\,\mathrm{capacity}\,\,[ \% ]=\frac{{{\rm{W}}}_{{\rm{wet}}}-{{\rm{W}}}_{{\rm{dwet}}}}{{{\rm{W}}}_{{\rm{dry}}}}$$

where, W_wet_ is the weight of the EO impregnated BC; W_dwet_ is the weight of the EO impregnated BC after centrifuging and W_dry_ is the dry weight of the BC before impregnation. The experiment was conducted in technical triplicates and repeated three times.

### Analysis of antimicrobial properties

The antimicrobial activity of impregnated BC was tested against two biofilm-forming bacteria namely *Staphylococcus aureus* (American Type Culture Collection - ATCC 6538) and *Pseudomonas aeruginosa* (ATCC 15442).

#### Agar diffusion disc method

The BC pellicles impregnated with EOs were placed onto the surface of the Mueller-Hinton (M-H) agar medium (BioMaxima, Poland) seeded with the suspension of *S*. *aureus* and *P*. *areuginosa* at a density of 1.5 × 10^8^ CFU/mL. Then, the cultures were carried out for 24 h at 37 °C. The average diameters of the inhibition zone (in mm) were calculated for each tested sample. The tests were performed in triplicate.

#### Well diffusion method

This standard method of EO antimicrobial activity assessment was performed exactly as described by Balouiri *et al*.^41^. The tests were performed in triplicate.

#### Anti-biofilm activity of impregnated BC – Antibiofilm Dressing Activity Measurement (A..D.A.M) test

To analyze the anti-biofilm activity of the BC impregnated with EOs the A.D.A.M test was performed^40^. Briefly, the strains from the Brain Heart Infusion (BHI) agar medium were transferred into 5 mL of Tryptic Soy Broth (TSB) medium and incubated for 24 h at 37 °C. On the next day, agar-filled wells of a 24-well plate were prepared. Agar discs of 2.5 mm in diameter were cut out from the Muller-Hinton agar plate, transferred to the fresh 24-well plate and immersed in 2 mL of ca. 10^5^ CFU of bacterial suspension. The discs with the bacterial suspension were incubated for 24 h at 37 °C. After incubation, the discs were rinsed 3 times with 2 mL of 0.9% NaCl to remove non-adherent bacteria and transferred to the agar-filled wells of the 24-well plate. Next, three discs (B - bottom, M - middle and C - contact) were placed into the holes. The BC membranes saturated with EOs were placed directly on the C-disc. On the next day, the MTT reduction cell viability assay was performed^40^.

The results are shown as % of living cells in the presence of BC impregnated with EOs, compared to pure cellulose (negative control) calculated by the following formula (2):

2 $${\rm{Viable}}\,{\rm{bacteria}}\,[ \% ]=(\frac{{{\rm{Abs}}}_{{\rm{IBC}}}-{{\rm{Abs}}}_{{\rm{B}}}}{{{\rm{Abs}}}_{{\rm{NC}}}-{{\rm{Abs}}}_{{\rm{B}}}})\,\ast \,100 \% $$

where, Abs_IBS_ is the absorbance measured in samples treated with BC impregnated with EOs; Abs_NC_ is the absorbance measured in a negative control (samples treated with pure BC); Abs_B_ is the absorbance of a blank sample (medium). The tests were performed in triplicate.

#### Penetrability and Penetrability Index [PI] calculation

The penetrability of the EOs released from an active dressing was determined after calculation of the efficacy for each disc (C, M, B) and after establishing the relationship between them based on the following assumptions:

- C~M~B - the EO displays high penetrability.
- C > M~B or C~M > B - the EO displays moderate penetrability.
- C > M > B - the EO displays weak penetrability.

Penetrability Index [PI] value was developed specifically for the A.D.A.M. test^40^. The formula for PI = C/B - (C/M)/(M/B); where C, B, M refer to the efficacy of biofilm reduction. PI value ≤ 1 describes dressings whose active substance displays good tolerability; PI <1> 2 describes dressings whose active substance displays moderate tolerability; PI ≤ 2 describes dressings whose active substance displays poor tolerability^40^.

### Antimicrobial activity of BC dressings against biofilmic forms of pathogens in an environment simulating bone conditions

#### Preparation of hydroxyapatite discs

Hydroxyapatite (HA) discs were used as a surface for growth of biofilm under conditions imitating bone environment. A commercially available HA powder was used for custom disc manufacturing. The subsequent stages of disc manufacturing were performed in the same manner as described in detail in our other papers^42–44^.

#### Scanning Electron Microscopy analysis of microbial biofilm formed on hydroxyapatite discs

Microbial dilutions were incubated on HA discs for 24 h at 37 °C. Next, the surfaces were rinsed 3x with 0.9% NaCl to remove non-adherent, loosely bound bacteria and to leave only biofilm forms of pathogens. Next, the samples were fixed and analyzed using Zeiss EVO MA25 scanning electron microscope (SEM) (Carl Zeiss, Germany) as described in our earlier study^31^.

#### Antimicrobial activity assessment under bone-imitating conditions

2 mL of 2% bacteriological agar were introduced to a well of a 24-well plate and left to consolide. Afterwards, an agar stake of 8 mm in diameter was cut out of agar using a cork-borer device (Conbest, Poland). The following experimental setting was performed to imitate bone environment: a HA disc (hydroxyapatite is the main inorganic component of bone) covered with *P*. *aeruginosa* or *S*. *aureus* biofilm was placed at the bottom of an agar-surrounded well and the empty space was filed with a medium for osteoblasts culture (F12 medium, Sigma-Aldrich, Germany) but without antimicrobials (antibiotics and antimicotics) which are introduced when a routine cell culture is performed. On the top of the agar-filled well, cellulose dressings saturated with EOs or saturated with polyethylene glycol (control samples - CP) were placed. The plates were incubated for 24 h at 37 °C in micro-aerobic conditions (5% CO_2_).

Next, the discs were subjected to 1 min of vigorous vortex-shaking in the presence of 0.1% saponine. Subsequently, the obtained suspension was serially diluted and cultured on Columbia Agar plate (in the case of *S*. *aureus*) or McConkey Agar plate (in the case of *P*. *aeruginosa*). The plates were incubated for 24 h/37 °C. After incubation, the number of colonies grown was counted. The results are shown as % of Biofilm Survival Rate calculated by the formula (3):

3 $${\rm{Biofilm}}\,{\rm{Survival}}\,{\rm{Rate}}\,\,[ \% ]=100 \% -(\frac{{{\rm{CFU}}}_{{\rm{EO}}}}{{{\rm{CFU}}}_{{\rm{CP}}}}\,\ast \,100 \% )$$

where, CFU_EO_ is a number of CFU survived treatment with specific EO; CFU_CP_ is a number of CFU grown in CP experimental setting. The tests were performed in triplicate.

#### Assessment of EO release from BC dressings and their binding to HA discs’ surface

BC discs soaked with EOs were placed in agar holes (prepared as described in *Antimicrobial activity assessment* section) with a HA disc inside. The entire experimental setting was covered with a plate lid and incubated for 24 h. Next, the HA discs were removed and washed in 1 ml methanol ChromaSolv (Sigma Aldrich, Poland) and dried under anhydrous sodium sulfate. 100 microliters of dry extracts were moved to glass inserts (250 µl) and injected directly on to a GC/MS system in splitless mode. The GC/MS method was described in the *Essential oils* section.

### Assessment of cytotoxicity of EO-saturated BC

The cytotoxicity activity of EO-impregnated BC was tested using Neutral red (NR) uptake, 3-(4,5-dimethylthiazol-2-yl)-2,5-diphenyl-2H-tetrazolium bromide (MTT) reduction and Alamar blue (AB) reduction assays in osteoblast (U2-OS) and fibroblast (L929) cell cultures treated with extracts obtained by incubation of EO-saturated BC membrane in the culturing medium. The extracts were prepared according to the ISO 10993 standard: Biological Evaluation of Medical Devices; Part 5: Tests for *In Vitro* Cytotoxicity; Part 12: Biological Evaluation of Medical Devices, Sample Preparation and Reference Materials (ISO 10993-5:2009 and ISO/IEC 17025:2005) using exactly the same protocol as we described already in our earlier reports^45^. The absorbance (NR and MTT) or fluorescence (AB) value of cells treated with PEG-containing extracts (CP sample) was considered 100%. The results are shown as % of Survival Rate calculated by the formula (4):

4 $${\rm{Survival}}\,{\rm{Rate}}\,[ \% ]=100 \% -(\frac{{\rm{Abs}}/{{\rm{Fl}}}_{{\rm{CP}}}-{\rm{Abs}}/{{\rm{Fl}}}_{{\rm{B}}}}{{\rm{Abs}}/{{\rm{Fl}}}_{{\rm{EO}}}-{\rm{Abs}}/{{\rm{Fl}}}_{{\rm{B}}}}\,\ast \,100 \% )$$

where, Abs/Fl_CP_ is the absorbance/fluorescence measured in cultures incubated with PEG-containing extracts; Abs/Fl_EO_ is the absorbance/fluorescence measured in cultures incubated with extract obtained from EO-saturated BC. Abs/Fl_B_ is the absorbance/fluorescence of a blank sample (medium). The tests were performed in triplicate.

### Macrophage ROS production in the presence of extracts from BC dressings impregnated with EOs

Immortalized bone marrow-derived macrophages (BMDM)^46^ were routinely cultured in DMEM medium (Biochrom, Germany) supplemented with 10% heat inactivated fetal bovine serum (Biowest SAS, France), l-Glutamine (4 mM, Biowest SAS), Penicillin-Streptomycin mix (100× dilution, Biowest SAS) and maintained in a humidified incubator at 37°C with 5% CO_2_ (CB-160, Binder, Germany). Reactive oxygen species (ROS) were measured with CellROX®Green (Thermofisher Scientific, USA), a cell-permeant dye that after penetration into cells in a reduced form exhibits weak fluorescence and when oxidized by intracellular free radicals, binds to DNA, emitting a more intense green fluorescence. For this assay, BMDM cells were plated on a 96-well plate at a final density of 1 × 10^5^ cells/well and incubated at 37 °C for 24 h in a CO_2_ incubator. In the next step, the cells were stained with CellROX®Green (final concentration of 5 µM) for 30 min at 37 °C with slight shaking. Then CellROX®Green was removed and the cells were treated with 10 µL of extracts for 1 h at 37 °C with slight shaking followed by fixation with 3.7% formaldehyde (POCH) in PBS and staining with DAPI (Sigma Aldrich, Germany) to visualize nuclei (0.5 µg/mL, 20 min, room temperature). The sample referred to as the “CP” which consisted of cellulose and oil PEG but did not possess active substances of EOs was considered a positive control. Another control setting aimed to show macrophage reactivity was lipopolysaccharide (LPS) isolated from *Escherichia coli* O55:B5 (2 ng/mL). Macrophages were examined on a spinning disk confocal microscope (Cell Observer SD, Zeiss, Germany) with a 20× dry objective (NA 0.4) and EMCCD Rolera EM-C2 camera (QImaging, Canada). In each experimental condition four random areas per well were chosen and confocal Z stacks of 14-bit images spaced at 3–5 µm were automatically acquired using Tiles mode of the Zeiss ZEN2 acquisition software. Image analysis was performed using standard operations in the Fiji software. To quantify ROS generation, maximum intensity projections from Z stacks were created and background noise was removed with Subtract Background function (radius 50). Next, DAPI channel was segmented and binarized to visualize nuclei together with cell bodies, which were identified due to the small amount of cytoplasm around the nuclei (fluorescence intensity threshold was typically 750–880). Small artifacts were removed with Median filter (radius 2) and clusters of cells were separated with Watershed function. Cell bodies were detected with Analyze Particles function (size between 44.5–445 µm^2^) and transferred onto the CellROX®Green channel in order to quantify ROS mean fluorescence intensity. Objects touching image edges were excluded from the analysis. The results were normalized with regard to CP sample which was assumed as a threshold of 100% value. The tests were performed in triplicate.

### Antioxidant effectiveness of extracts obtained from BC impregnated with essential oils

Antioxidant effectiveness of extracts obtained from BC impregnated with EOs was examined by two different radical scavenging assays: 2,2-diphenyl-1-picrylhydrazyl (DPPH) and 2,2′-azinobis-3-ethylbenzothiazoline-6-sulfonic acid (ABTS). The extracts were prepared using exactly the same protocol as we described already in *Neutral red cytotoxicity assay* section.

#### 2,2′-Azinobis-3-ethylbenzothiazoline-6-sulfonic acid cation radical-scavenging

The measurement of 2,2′-azinobis-3-ethylbenzothiazoline-6-sulfonic acid (ABTS) (Sigma-Aldrich, Germany) radical scavenging was carried out according to the protocol of Rufino *et al*. (2010)^47^ with some modifications. The ABTS radical solution was prepared by mixing 7.0 mmol/L ABTS and 2.5 mmol/L of potassium persulfate. Extracts of 10 μL were subsequently mixed with 290 μL of ABTS radical solution, and the absorbance of the resulting mixtures was measured after 30 min at 735 nm using Infinite 200 PRO NanoQuant reader (Tecan, Männedorf, Switzerland). The free radical-scavenging capacity was calculated by the following Equation (5):

5 $${\rm{Radical}}\,{\rm{Scavenging}}\,[ \% ]=(\frac{{{\rm{Abs}}}_{{\rm{B}}}-{{\rm{Abs}}}_{{\rm{S}}}}{{{\rm{Abs}}}_{{\rm{B}}}})\,\ast \,100 \% $$

where, Abs_B_ is the absorbance of the ABTS mixed with deionized water; Abs_S_ is the absorbance of the ABTS mixed with extracts. All measurements were performed in triplicate and reported as the average value.

#### 2,2-Diphenyl-1-picrylhydrazyl free radical-scavenging capacity

The measurement of 2,2-diphenyl-1-picrylhydrazyl (DPPH) (Sigma-Aldrich, Germany) radical scavenging capacity was carried out according to Karamać *et al*.^48^ 290 μl of 0.5 mmol/L DPPH in methanol (Meyer, México) was mixed with 10 μL of the extracts. The mixture was incubated for 30 min at room temperature followed by absorbance reading at 417 nm using Infinite 200 PRO NanoQuant reader (Tecan, Männedorf, Switzerland). The percentage of free radical-scavenging capacity was calculated by the following Equation (6):

6 $${\rm{Radical}}\,{\rm{Scavenging}}\,{\rm{Capacity}}\,[ \% ]=\,(\frac{{{\rm{Abs}}}_{{\rm{B}}}-{{\rm{Abs}}}_{{\rm{S}}}}{{{\rm{Abs}}}_{{\rm{B}}}})\,\ast \,100 \% $$

where, Abs_B_ is the absorbance of DPPH mixed with methanol instead of with the extracts; Abs_S_ is the absorbance of DPPH mixed with the extract. All measurements were performed in triplicate and reported as an average value.

### Statistical analyses

Calculations were performed using the GraphPad Prism version 7 software. Normality distribution was calculated by means of D’Agostino-Pearson omnibus test. Because all values were non-normally distributed, the Kruskal-Walis test with post-hoc Dunnet analysis were applied. The results of statistical analyses were considered significant if they produced p-values < 0.05.

## Supplementary information

Supplementary information for the manuscript entitled Potential of Biocellulose Carrier Impregnated with Essential Oils to Fight Against Biofilms Formed on Hydroxyapatite

## Data Availability

All data generated or analysed during this study are included in this published article (and its Supplementary Information files).
