# Supplementary material for: Potential of Biocellulose Carrier Impregnated with Essential Oils to Fight Against Biofilms Formed on Hydroxyapatite
Source: Sci Rep. 2019 Feb 4;9:1256. doi: 10.1038/s41598-018-37628-x (PMC6362291; doi:10.1038/s41598-018-37628-x)
Supplement: Supplementary file 1 — Supplementary information for the manuscript entitled Potential of Biocellulose Carrier Impregnated with Essential Oils to Fight Against Biofilms Formed on Hydroxyapatite [file 41598_2018_37628_MOESM1_ESM.docx]

**Supplementary information**

**Potential of Biocellulose Carrier Impregnated with Essential Oils to Fight Against Biofilms Formed on Hydroxyapatite**

**Adam Junka^1^, Anna Żywicka^2^, Grzegorz Chodaczek^3^, Mariusz Dziadas^4^, Joanna Czajkowska^5^, Anna Duda-Madej ^6^, Marzenna Bartoszewicz^1^, Katarzyna Mikołajewicz^3^, Grzegorz Krasowski^7^, Patrycja Szymczyk^8^, Karol Fijałkowski^2*^**

^1^Department of Pharmaceutical Microbiology and Parasitology, Wrocław Medical University, Borowska 211A, 50-556 Wrocław, Poland, feliks.junka@gmail.com, m.bartoszewicz@op.pl

^2^Department of Immunology, Microbiology and Physiological Chemistry, West Pomeranian University of Technology, Szczecin, Piastów 45, 70-311 Szczecin, Poland, anna.zywicka@zut.edu.pl, karol.fijalkowski@zut.edu.pl

^3^Laboratory of Confocal Microscopy, Polish Center for Technology Development PORT, Stablowicka 147, 54-066 Wrocław, Poland, grzegorz.chodaczek@eitplus.pl, katarzyna.mikolajewicz@eitplus.pl

^4^Faculty of Chemistry, University of Wroclaw, Joliot-Curie 14, 50-353 Wrocław, Poland, mariuszdziadas@gmail.com

^5^Laboratory of Microbiology, Polish Center for Technology Development PORT, Stabłowicka 147, 54-066 Wrocław, Poland, joanna.czajkowska@eitplus.pl

^6^ Department of Medical Microbiology, Wroclaw Medical University, Chałubińskiego 4, 50-534 Wrocław, Poland, anna.duda-madej@umed.wroc.pl

^7^Nutrikon, KCZ Surgical Ward, Krakowska 32A, 46-020 Opole, Poland, g.krasowski@wp.pl

^8^ Centre for Advanced Manufacturing Technologies (CAMT/FPC), Faculty of Mechanical Engineering, Wrocław University of Science and Technology, Łukasiewicza 5, 50-371 Wrocław, Poland, patrycja.szymczyk@gmail.com

Correspondence and requests for materials should be addressed to Fijałkowski K. (email: [karol.fijalkowski@zut.edu.pl](mailto:karol.fijalkowski@zut.edu.pl))

**Supplementary information includes:**

**1. Supplementary figures**

**2. Supplementary figure legends**

**3. Supplementary table**

**4. Supplementary table legend**

| **A** | **B** | **C** |
| --- | --- | --- |
| 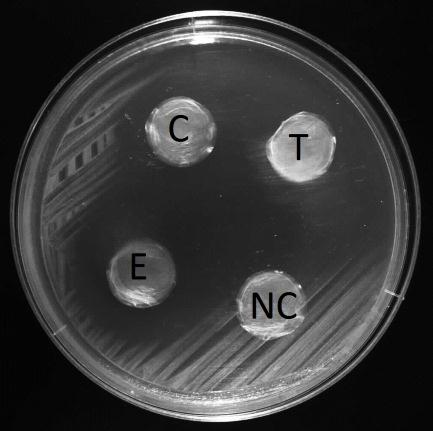 | 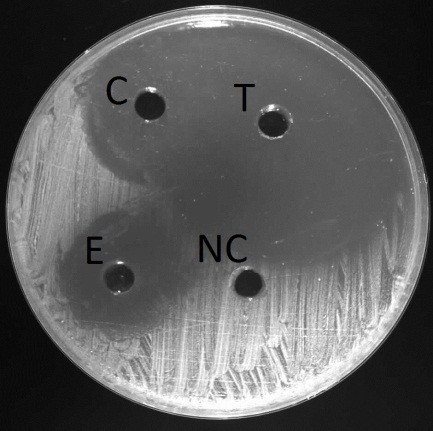 | 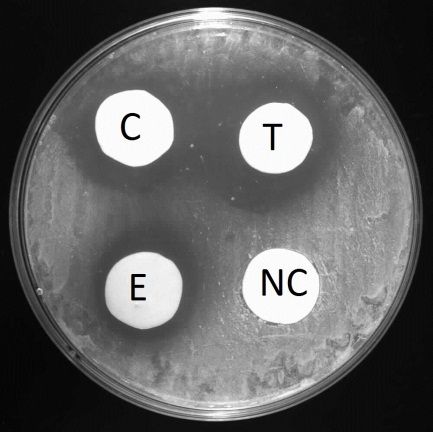 |

**Figure S1**. Antimicrobial activity of BC impregnated with EOs, EOs alone and EOs in paper discs (NC - negative control) against *S. aureus.* C - clove oil, E - eucalyptus oil, T - thyme oil.

| 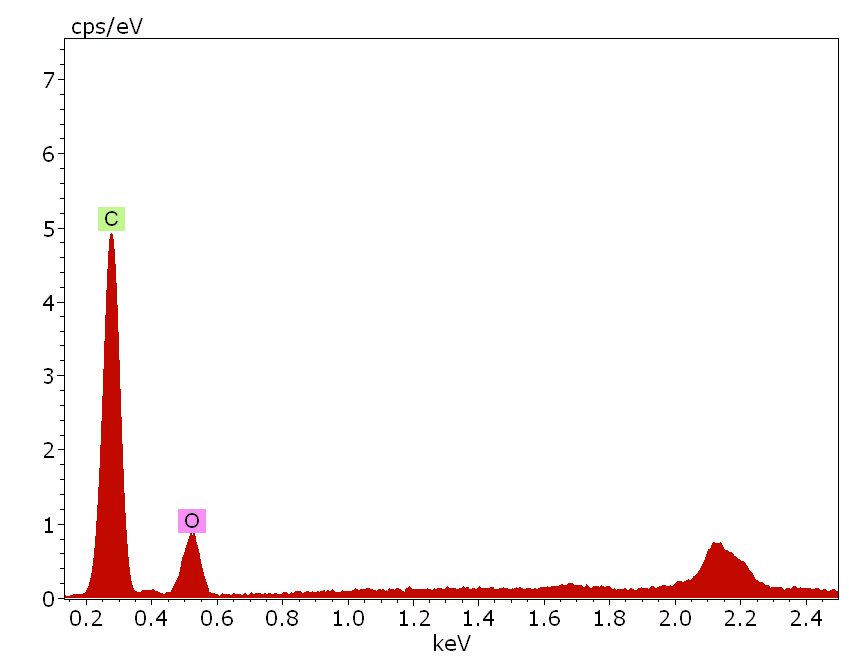 |  |
| --- | --- |

**Figure S2.** Elemental composition of BC formed by *K. xylinus* DSM 46602 after purification with NaOH and washing with water.

**Table S1.** Survival Rate [%] of fibroblasts incubated in presence of extracts from BC dressings impregnated with EOs.

|  | **AlamarBlue** | **MTT** |
| --- | --- | --- |
| **E-BC** | 95.5 | 73.2 |
| **C-BC** | 22.9 | 19.2 |
| **T-BC** | 21.6 | 25.8 |
| **PC** | 87.3 | 87.4 |
| **BC** | 100 | 100 |
| **Medium** | 104.6 | 127.7 |

where, C - clove oil, E - eucalyptus oil, T - thyme oil.; BC- bacterial cellulose; PC - bacterial cellulose impregnated with glycol.
